# Choroidal melanoma with synchronous Fuchs’ adenoma and novel *ATRX* mutation

**DOI:** 10.1186/s40942-022-00374-4

**Published:** 2022-04-01

**Authors:** Elli Harford, Jane M. Palmer, William J. Glasson, Sunil K. Warrier, Kevin J. Whitehead, Kelly M. Brooks, Peter A. Johansson, Nicholas K. Hayward, Lindsay A. McGrath

**Affiliations:** 1 Griffith University, School of Medicine, Brisbane, QLD Australia; 2 QIMR Berghofer Medical Research Institute, Brisbane, QLD Australia; 3 Queensland Ocular Oncology Service, Terrace Eye Centre, 2/87 Wickham Terrace, Spring Hill, QLD 4011 Australia; 4 Sullivan Nicolaides Pathology, Brisbane, QLD Australia; 5 University of Queensland, School of Medicine, Brisbane, QLD Australia

**Keywords:** Fuchs Adenoma, Uveal Melanoma, ATRX

## Abstract

**Background:**

To report a case of Fuchs’ adenoma occurring in an eye with a large choroidal melanoma. We have reviewed the literature to describe the clinical presentation, ultrasound characteristics and pathological features of these entities.

**Case presentation:**

A 69-year-old Caucasian man presented with vision loss from a large choroidal melanoma. Enucleation showed an incidental Fuchs’ adenoma in the same eye. Whole-exome sequence analysis was also performed on the patient’s blood and melanoma, which showed a rarely-reported *ATRX* mutation.

**Conclusions:**

Fuchs’ adenoma is an under-diagnosed benign age-related hyperplasia of the non-pigmented ciliary epithelium (NPCE). Given its location and characteristics, it can be mistaken for choroidal melanoma and clinicians are reminded how to differentiate between these pathologies and that they may co-exist.

## Background

First described by Fuchs in 1883 as a “defective pigment layer of the ciliary process…composed of cell strands embedded in a homogenous mass”, Fuchs’ adenoma is a benign tumor that develops at the pars plicata of the ciliary body [1, 2]. Also known as coronal adenoma or pseudoepitheliomatous hyperplasia, Fuchs’ adenoma is believed to be a reactive age-related hyperplasia of the non-pigmented ciliary epithelium (NPCE) with clinical onset in adulthood at a mean age of 45 years [2, 3]. Despite these lesions’ commonality, being found in 20–31% of eyes post mortem, Fuchs’ adenomas are rarely encountered on examination and only occasionally become large enough to assume clinical significance [4–6]. Severe adenoma of the NPCE can cause symptoms such as secondary cataract (80%), intraocular inflammation (40%), subluxation of the lens (40%), vitreous hemorrhage (15%), secondary glaucoma (15%) and cystoid macular edema (5%) [7].

Historically, due to their close clinical resemblance, Fuchs’ adenomas have been misdiagnosed as ciliary body or iris melanomas [8]. Reports by Shields [9], Zaidman^2^ and Nagarkatti-Gude [8] describe three cases where lesions of the ciliary body or peripheral iris were excised due to concern for malignant melanoma, that were subsequently found to be Fuchs’ adenoma [8]. Furthermore, it has been reported that Fuchs’ adenoma is generally found in eyes with a history of ocular abnormalities [4, 10]. Despite this, no diagnosis of Fuchs’ adenoma in a patient with uveal tract melanoma has been described [8]. Here, we report the first known case of concurrent choroidal uveal melanoma with Fuchs’ adenoma. A whole-exome genomic analysis is also included, with novel findings.

## Case presentation

A 69-year-old male was referred to the ocular oncology clinic with a 2-week history of reduced vision in his left eye. He had a history of type II diabetes, and a cutaneous melanoma excised from the right anterior chest 12 months previously. He was an ex-smoker for 15 years and had mild asthma and hypercholesterolemia. He had an unremarkable past ocular history, with minimal diabetic retinopathy noted in the contralateral eye.

**Fig. 1:**
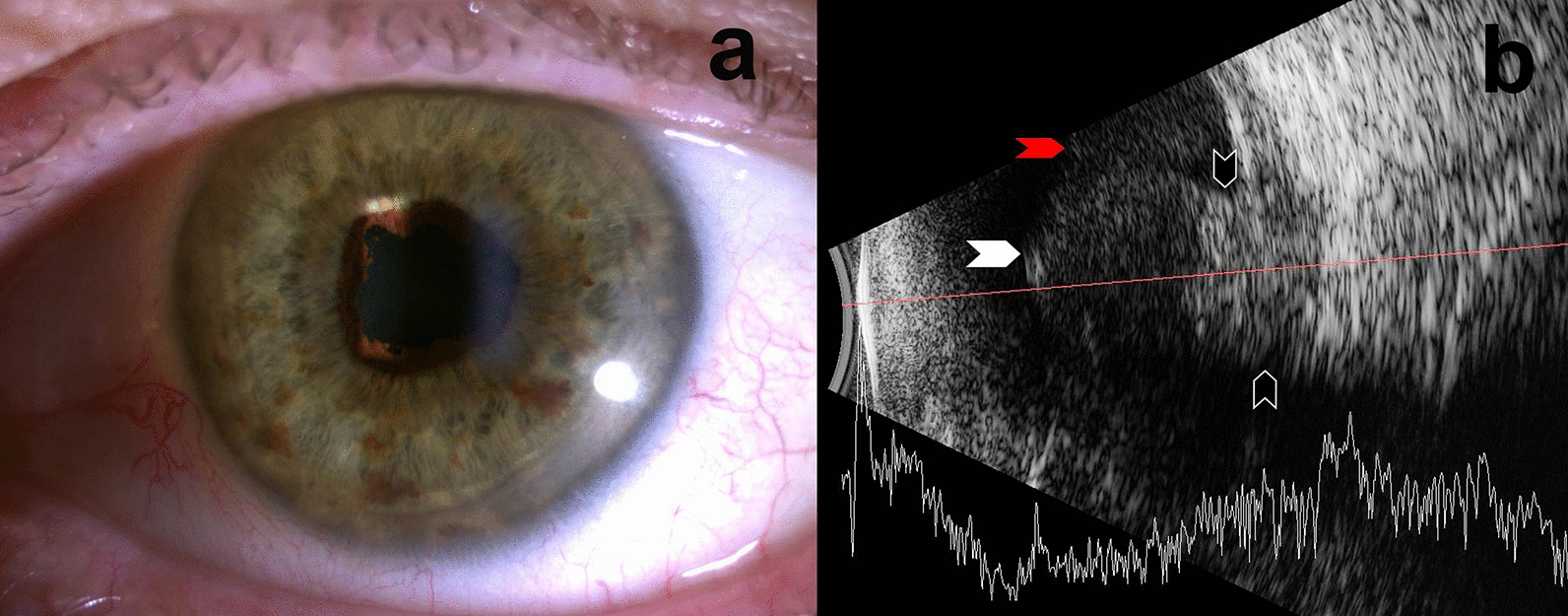
**a** Anterior segment with posterior synechiae and cataract. **b** Bscan ultrasound showing posterior segment choroidal mass. Red Arrow: Vitreous hemorrhage, White Arrow: Anterior edge of tumor, Black Arrows: Collar-stud base

On examination, visual acuity was 20/10 in OD and light perception in OS. Intraocular pressure was normal in both eyes. There was moderate left anterior uveitis with 360 degrees of posterior synechiae and moderate cataract (Fig. 1a). There was a dense vitreous hemorrhage and no fundus view. Ultrasound showed a 14 × 10 mm mass at 11:00, with low internal reflectivity and a collar-stud appearance (Fig. 1b). The apical thickness was measured at 9 mm. The vitreous hemorrhage was attributed to a break in Bruch’s membrane.

## Pathological findings

**Fig. 2:**
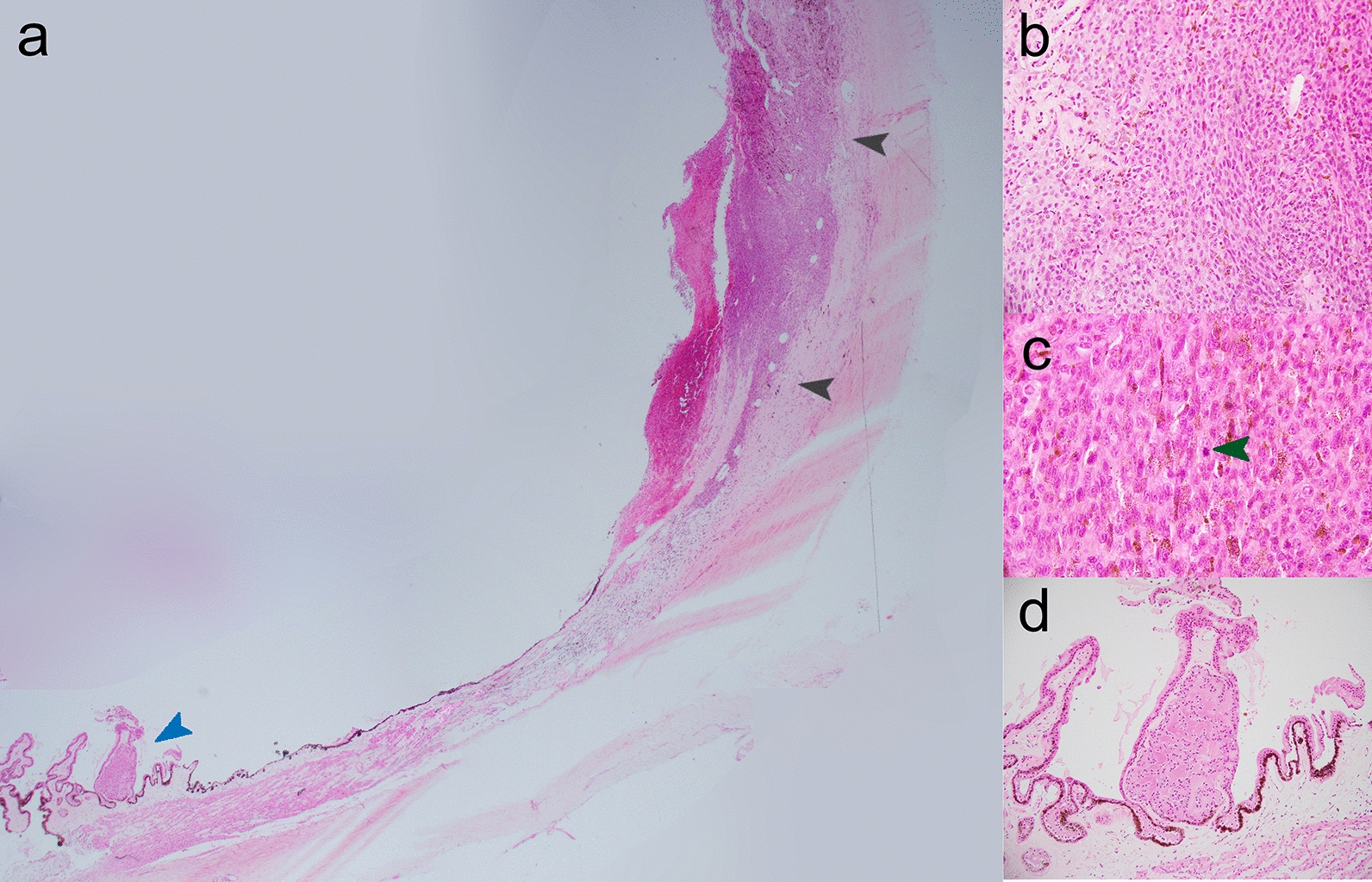
Enucleation specimen left eye, ciliary body and anterior choroid. Haematoxylin and eosin (H&E) × 4. **a** Blue Arrow: Fuchs’ Adenoma, Black Arrows: Choroidal Melanoma. **b** Choroidal melanoma (× 20 magnification). **c** Choroidal melanoma, Green Arrow: Mitotic Figure (× 40 magnification). **d** Fuchs’ Adenoma isolated (× 10 magnification)

Management options including plaque brachytherapy and enucleation were discussed. The patient proceeded to left enucleation due to guarded visual prognosis, size of the mass and clinical suspicion of uveal melanoma. Macroscopically, there was a brown nodule measuring 13 × 11 × 7 mm in the superior wall of the globe. Histopathology confirmed choroidal melanoma with uniform spindle B cytomorphology and no tumor necrosis. There was limited direct invasion of scleral connective tissue; however, there was extension along emissary structures through the almost the entire thickness of the sclera, although no epibulbar deposits were seen. The tumor base extended from the pars plana anteriorly to the post-equatorial zone posteriorly, falling well short of the optic nerve head. The optic nerve resection margin was tumor free. No involvement of the posterior chamber or anterior chamber was seen, and no tumor emboli were seen in sections of vortex veins. A Fuchs’ adenoma was also seen as an incidental feature (Fig. 2).

To further characterize genomic changes in the melanoma, we performed whole-exome sequence analysis. DNA was extracted from the melanoma and blood samples using AllPrep DNA/RNA Kit (80204, Qiagen Ltd, Hilden, Germany) and standard salting-out methods, respectively. Sequencing libraries were constructed using SureSelect V7-Post and sequencing was performed on the Illumina platform by Macrogen (Seoul, South Korea). Sequence reads were aligned against the human reference genome (humanG1Kv37) and mutations were identified as previously described [11]. Only four nonsynonymous somatic mutations were observed in the melanoma. The tumor harbored a GNAQ:p.Q209P mutation, a frequently occurring and known driver mutation in uveal melanoma [12]. A missense (p.L303P) mutation of unknown significance was seen in *RB1. BAP1* had a missense (p.S63C) mutation, which previously has been observed as a germline variant in mesothelioma patients [13]. BAP1 staining revealed loss of nuclear protein, but the loss is probably not caused by the missense mutation, as the p.S63C-mutated protein retains the function of the wildtype protein [14]. A splice site variant (c.4121-1G > T) was identified in *ATRX*, truncating the only copy of the known tumor suppressor located on chromosome X. Cytogenic analysis revealed no abnormalities in chromosome 3, but increase in copy number of 8q was detected.

Routine initial diagnostic screening whole body positron emission computerized tomography (PET/CT) was suspicious for liver metastases in segment VI and VII. The patient elected to proceed with enucleation for primary tumour management, despite these findings. Magnetic resonance imaging of the liver for further delineation confirmed multiple liver lesions including dominant lesions in segment VI and VII measuring up to 11.3 cm in diameter, with extension into the proximal right hepatic vein and inferior vena cava. A separate satellite lesion measuring 43 mm was noted in segment VI and several other small lesions involving both the right and left hepatic lobes were also seen. Fine needle aspirate of one of the larger lesions confirmed metastatic melanoma. Liver function testing revealed mild hepatic dysfunction and a lactate dehydrogenase (LDH) of 500. The patient passed away 8 months after presentation due to metastatic melanoma.

## Discussion

Adenomas of the NPCE are acquired amelanotic tumors commonly found in the eyes of older individuals, but they rarely grow large enough to become clinically apparent or misdiagnosed as malignant iris or ciliary body melanoma [15]. In our case, the NPCE adenoma was very anterior, and small, and was not visible clinically or detected on ultrasound. This is in keeping with previous authors’ nomenclature of these lesions as idiopathic, senile hyperplasia of the NPCE [16]. In some cases, Fuchs’ adenoma is detected if it displaces the iris anteriorly or passes through the peripheral iris, potentially causing secondary focal cataract and subluxing the lens, raising suspicions of melanoma. A study by Shields et al. identified characteristic features that serve to differentiate tumors of the NPCE from ciliary body melanoma [4]. They describe that Fuchs’ adenoma is located internally to pigment epithelium, whereas melanoma is located in the uveal stroma external to ciliary pigment epithelium, resulting in a mildly pigmented surface. In general, they appear as a white mass in the pars plicata, confined to one ciliary process. Despite this, cases of larger, variably pigmented adenomas of the NPCE (due to extreme iris stromal atrophy) have been reported by Shields and Nagarkatti, suggesting that this presentation may be more common than previously recognized [8]. Additionally, while some cases of Fuchs’ adenoma may present with inflammation within the anterior chamber and sentinel vessel in the overlying episclera, this is more common in uveal melanoma. Fuchs’ adenomas are less likely to have sentinel vessels and are more likely to show abrupt elevation, acoustic solidity and high internal reflectivity due to cystic spaces arising from lacunar tissue defects. The current case report highlights that Fuchs’ adenoma may also show minimal clinical signs until they are of significant size, in the same way that melanomas of the ciliary body present later due to fewer symptoms than their posterior uveal counterparts.

Microscopically, as in this case, Fuchs’ adenoma is composed of convoluted sheets of the NPCE containing type IV collagen and laminin, between which are varying amounts of amorphous, eosinophilic PAS-positive extracellular material. Multiple studies have confirmed the strong immunoreactivity of Fuchs’ adenoma cells to S-100 moderate immunoreactivity to vimentin and cytokeratin, with negative reactivity to melanoma-specific HMB45, confirming the origin as nonpigmented ciliary epithelial [4, 7]. Electron microscopy shows intercellular interdigitations with numerous desmosomes, consistent with cells of the NPCE [17]. Fifty years ago, the polymorphism of these groups of tumors was emphasized by Zimmerman and colleagues [18]. In particular, the spectrum of adenoma to epithelioma is exemplified by the variable presence of hyaluronidase-sensitive mucopolysaccharide (identical to vitreous) associated with the tumor [18]. Fuchs’ Adenoma is a solid tumor with cellular proliferation in the form of papillae and clusters that grow on the internal surface of the ciliary body without invading the stroma—unlike melanoma of the same region [3].

Acquired epithelioma of the NPCE is another important differential which has benign and malignant subtypes [17]. These lesions can be associated with mild intraocular inflammation. They can have significant vascularity, and may present with vitreous hemorrhage. Tumors of the NPCE, in contrast to melanoma, usually transmit light on transillumination. An additional factor distinguishing NPCE neoplasms from ciliary body melanoma is that tumors of the NPCE may have a more irregular surface with cells of the tumor arranged in linear bands along the septa of the extra-cellular matrix material [3, 7].

Over 20 cases of acquired adenoma of the NPCE have been reported in English literature and can be distinguished from adenocarcinoma of the NPCE, or melanoma, by the absence of rare mitoses and local infiltrative behaviour [19].

On genetic analysis of the concurrent melanoma in this case, a GNAQ:p.Q209P mutation was found, which is a known driver in uveal melanoma. More interestingly, a splice site variant (c.4121-1G > T) was identified in *ATRX*. Loss-of-function mutations in *ATRX* are frequently seen in other subtypes of melanoma but is not typically seen in uveal melanoma [20]. The American Association for Cancer Research recently reported an *ATRX* loss-of-function mutation in one uveal melanoma in their cancer registry [21]. This inactivation is commonly associated with alternative lengthening of telomeres which overrides telomere maintenance driven by cellular immortality mechanisms. In turn, this can lead to cellular crisis and genomic instability.

## Conclusion

Although common in enucleation specimens, clinical diagnosis of Fuchs’ adenoma remains rare despite advances in anterior segment imaging. We report a case of Fuchs’ adenoma occurring in an eye with advanced choroidal malignant melanoma. The diagnostic workup of uveal melanoma includes ultrasound imaging, and it is useful for clinicians to be reminded that these lesions can co-exist.

## Data Availability

Not Applicable.
